# Brentuximab vedotin use in pediatric anaplastic large cell lymphoma

**DOI:** 10.3389/fimmu.2023.1203471

**Published:** 2023-05-18

**Authors:** Jennifer E. Agrusa, Emily R. Egress, Eric J. Lowe

**Affiliations:** ^1^ University of Michigan, Division of Pediatric Hematology-Oncology, Ann Arbor, MI, United States; ^2^ Eastern Virginia Medical School, Department of Pediatrics, Norfolk, VA, United States; ^3^ Division of Pediatric Hematology-Oncology, Children’s Hospital of The King's Daughters, Norfolk, VA, United States

**Keywords:** Brentuximab vedotin, anaplastic large cell lymphoma, ALCL, CD30, non-Hodgkin lymphoma, pediatric

## Abstract

Anaplastic large cell lymphoma (ALCL) is the most common type of mature T-cell non-Hodgkin lymphoma in children/adolescents. ALCL is characterized by expression of CD30 in the neoplastic lymphoid cells with frequent expression of anaplastic lymphoma kinase (ALK), especially within the pediatric population. Despite multiple efforts to optimize the use of conventional chemotherapy, outcomes in children, adolescents, and adults with ALCL remain suboptimal. Thus, there is a need to improve survival for those with high-risk disease and decrease therapy exposures and toxicities for those with low-risk disease. Targeted therapies, such as the anti-CD30 antibody-drug conjugate, brentuximab vedotin, are new important therapeutic options. Phase I and II studies in adults with relapsed/refractory CD30^+^ lymphomas, including ALCL, demonstrated the safety and efficacy of brentuximab vedotin, leading to FDA approval for relapsed/refractory ALCL in adults and successful incorporation into frontline therapies. Clinical trials in the pediatric population demonstrated similar results in those with relapsed/refractory ALCL. Incorporation of brentuximab vedotin into upfront therapy for children and adolescents with ALCL showed that this novel combination therapy has clinical advantages in comparison to conventional agents alone. Brentuximab vedotin is well-tolerated in both the pediatric and adult populations, even when used in combination with conventional agents. Brentuximab vedotin is an ideal agent to treat ALCL with excellent targeted activity and limited toxicity. Future studies are needed to identify how brentuximab vedotin should be utilized when combined with immunotherapy or other targeted agents (e.g., ALK inhibitors) in both the upfront and relapsed/refractory setting.

## Introduction

Anaplastic large cell lymphoma (ALCL) is a type of non-Hodgkin lymphoma (NHL) that is characterized by a neoplastic proliferation of CD30^+^ lymphoid cells, with frequent expression of anaplastic lymphoma kinase (ALK) (1, 2). While ALCL affects only 2% of adults with NHL, it comprises 15-20% of all pediatric NHLs (3–5). The major forms of ALCL include primary cutaneous ALCL, which is primarily seen in adults and is usually ALK^-^, and systemic ALCL, which can be ALK^+^ or ALK^-^ (6). In contrast to ALCL in adults, the majority of ALCLs in pediatrics are ALK^+^ (60% vs. 80-95%) and involve a translocation that fuses the *nucleophosmin (NPM)* and *anaplastic lymphoma kinase (ALK)* genes (7–10).

While the ALK-fusion protein is inconsistently expressed in ALCL, CD30 (also known as Ki-1) is a cell membrane protein that is identified in all cases. The features that differentiate ALCL from other types of CD30^+^ malignancies include co-expression of CD30 with epithelial membrane antigen and/or T cell antigens in the presence of anaplastic morphologic features (1, 2). CD30 is part of the tumor necrosis factor (TNF) receptor superfamily, specifically TNF receptor superfamily member 8 (TNFRSF8). The universal expression of CD30 in systemic ALCL provides an opportunity to provide targeted therapy utilizing this unique target (11–14). For this reason, the chimeric anti-CD30 antibody-drug conjugate, brentuximab vedotin, has become an important therapeutic option in treatment of ALCL.

## Targeting CD30 expression with brentuximab vedotin

### Mechanism of action

**Figure 1:**
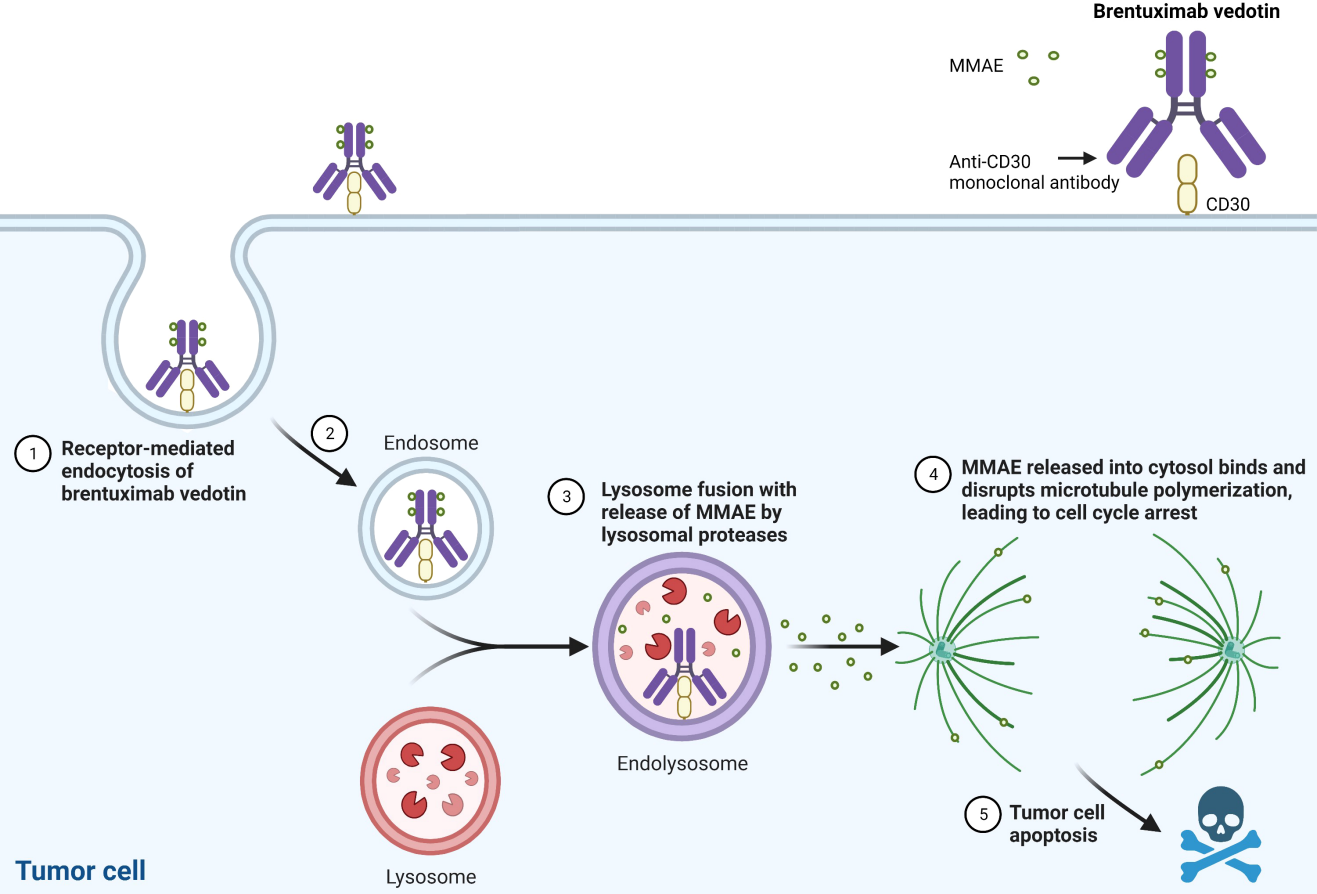
Brentuximab Vedotin’s Mechanism of Action. MMAE: monomethyl auristatin E. Created with BioRender.com.

Brentuximab vedotin is generated by conjugating the anti-CD30 monoclonal antibody, SGN-30, to monomethyl auristatin E (MMAE), a synthetic anti-neoplastic agent that binds tubulin. SGN-30 demonstrated both *in vitro* and *in vivo* efficacy in CD30^+^ lymphoma (15), and the potency was increased by linking it to MMAE (16). Brentuximab vedotin binds to the surface antigen CD30, undergoes lysosomal internalization, and releases MMAE into the cytosol, thereby inhibiting microtubule polymerization and inducing apoptosis (**Figure 1**). Aside from direct tumor cell cytotoxicity by MMAE, preclinical studies suggest that brentuximab vedotin exhibits additional mechanisms of action, including: 1) bystander killing, in which MMAE exerts toxicity on adjacent tumor cells, 2) immunogenic cell death by inducing dendritic cell maturation and activating a cellular immune response and/or by inducing endoplasmic reticulum stress due to the disruption of the microtubule network, and 3) antibody-dependent cellular phagocytosis mediated by macrophages (17–22).

### Preclinical data

Prior to the development of brentuximab vedotin, unconjugated anti-CD30 monoclonal antibodies demonstrated *in vitro* activity against ALCL. Incubation of two CD30-expressing ALCL cell lines with two anti-CD30 monoclonal antibodies (M44 or HeFi-1) resulted in significant growth inhibition and decreased cell viability (23). Mice injected with these ALCL cell lines developed various metastases, but treatment with anti-CD30 monoclonal antibodies resulted in improvement in survival.

When conjugating CD30 to MMAE (cAC10-vcMMAE), preclinical studies showed that the conjugated drug was highly potent and selectively more active against CD30^+^ cells in comparison to CD30^-^ cells (16). The antibody drug conjugate also demonstrated clinical activity against mice xenograft models of ALCL or Hodgkin lymphoma treated with doses of 1 – 30 mg/kg without signs of toxicity. The efficacy of vinblastine in relapsed/refractory ALCL established precedence for the use of tubule inhibition, and preclinical data demonstrated higher potency using brentuximab vedotin versus vinblastine against CD30^+^ cell lines (24–26).

## Conventional therapy for ALCL

### Adults

The rarity of systemic ALCL has made effective therapy modalities difficult to study in adults, as this disease is often included with other forms of peripheral T cell lymphomas. Prior to the development of brentuximab vedotin, regimens that included cyclophosphamide, anthracycline, vincristine, and prednisone (CHOP) or CHOP-like regimens were used most frequently. In a systematic review/meta-analysis of anthracycline-based chemotherapy for peripheral T cell lymphoma, the complete remission (CR) rate for ALCL was 65.8% with a 5-year overall survival (OS) of 56.5% (27). The 2-year event-free survival (EFS) was no different between CHOP and another regimen VIP-reinforced-ABVD (etoposide, ifosfamide, cisplatin alternating with doxorubicin, bleomycin, vinblastine, dacarbazine) (28). Adding etoposide to CHOP (CHOEP) improved the progression-free survival (PFS) in patients with peripheral T cell lymphoma, especially if ≤60 years old and in those with ALK^+^ ALCL (hazard ratio [HR] for ≤60 years old = 0.49, P=.008) (29, 30). Autologous stem cell transplants were also considered in adults with ALCL, though the lack of randomized clinical trials precluded demonstration of any specific survival advantage (31). Prior to the availability of brentuximab vedotin, the treatment options for adults with systemic ALCL were limited and toxic (29, 32, 33).

### Pediatrics

Similar to adults, outcomes for children/adolescents with systemic ALCL were suboptimal with a failure rate of 25 – 30% despite multiple attempts to improve upfront therapy. Adding methotrexate and high-dose cytarabine to APO (doxorubicin, prednisone, and vincristine) chemotherapy, compressing the duration of T-cell lineage therapy, or adding vinblastine to APO produced increased toxicities without improving efficacy (34–36). In the largest pediatric clinical trial to date with 353 patients, the ALCL99 study (prophase of dexamethasone and cyclophosphamide followed by 6 alternating courses (Courses 1, 3, 5: dexamethasone, methotrexate, ifosfamide, cytarabine, etoposide, +/- vinblastine and Courses 2, 4, 6: dexamethasone, methotrexate, cyclophosphamide, doxorubicin, +/- vinblastine) given every 21 days) showed no advantage for the use of methotrexate given over 24 hours with intrathecal chemotherapy or for the addition of weekly vinblastine in maintenance (24).

Outcomes for those with relapsed ALCL, and especially for patients who progress on therapy, are also suboptimal. Brugieres et al. performed a retrospective study within a heterogeneous population of patients with relapsed/refractory ALCL who received vinblastine salvage therapy and found that the 5-year EFS was only 30% (37). Chemotherapy followed by hematopoietic stem cell transplant (HSCT) consolidation is another option with one study demonstrating a 5-year EFS of 59% (38). While Gross et al. found no significant difference in 5-year EFS when comparing autologous (35%) versus allogeneic (46%) HSCT, Knorr et al. found that allogeneic HSCTs may be a more effective consolidation for those with early relapse (26, 39). Prior to the availability of brentuximab vedotin and ALK inhibitors, patients who progress on therapy have an EFS of only 25 – 41%, even with HSCT (26, 38, 40, 41). Given the high relapse rate and poor prognosis for those who do relapse or progress on therapy, targeted agents are promising therapeutic options for these patients.

## Clinical efficacy of brentuximab vedotin in adults

### Relapsed/refractory therapy

Several studies have evaluated the safety and clinical efficacy of targeting CD30^+^ lymphomas using brentuximab vedotin (**Table 1**). A phase I dose-escalation study of brentuximab vedotin enrolled heavily pre-treated patients with relapsed/refractory CD30^+^ hematologic malignancies (Hodgkin lymphoma, n=42; systemic ALCL, n=2) (42). The maximum tolerated dose was 1.8 mg/kg administered every three weeks, and the CR rate at this dose was 100% for the two patients with ALCL. Most adverse events (AEs) were mild (grades 1 – 2), and included fatigue, pyrexia, diarrhea, nausea, neutropenia, and peripheral neuropathy. The only grade 3 AE at the maximum tolerated dose was neutropenia (n=1) and pain (n=2).

**Table 1:** Studies evaluating brentuximab vedotin in anaplastic large cell lymphoma (ALCL).

Treatment	Adult or Ped	Relapsed/Frontline	Phase	# ALCL Patients	Median Age (years)	ALK+	Key Findings	Ref
Brentuximab	Adult	Relapsed/Refractory	I	2	36 (of all pts, including with HL)	2/2 (100%)	Both achieved CR	(42)
Brentuximab	Adult	Relapsed/Refractory	II	58	52	16/58 (27.6%)	OR 86% CR 66%	(43)
Brentuximab	Adult	Relapsed/Refractory	I	5	33	1/5 (20%)	CR 80%	(44)
Brentuximab (after previous treatment with brentuximab)	Adult	Relapsed/Refractory	II	8	33	3/8 (37.5%)	OR 88%, CR 63%	(45)
Sequential w**/**CHOP vs Combination (A+CHP)	Adult	Frontline	I	32	57	6/32 (18.8%)	Seq: OR 85%, CR 62%, 1-year PFS 77% Comb: OR 100%, CR 88%, 1-year PFS 71%	(46)
A+CHP vs CHOP	Adult	Frontline	III	316	58	98/316 (31%)	A+CHP: 5-yr PFS 51.4%, OS 70.1% CHOP: 5-yr PFS 43.0%, OS 61.0%	(47)
Brentuximab	Pediatric	Relapsed/Refractory	I/II	17	14 (of all pts, including with HL)	12/17 (70.6%)	OR 53%, CR 41%	(48)
ANHL12P1 Arm BV	Pediatric	Frontline	II	68	12	68/68 (100%)	2-yr EFS 79.1%, 2-yr OS 97.0%	(40)

HL, Hodgkin Lymphoma; CR, Complete Response; OR, Objective Response; EFS, Event-Free Survival; PFS, Progression Free Survival; OS, Overall Survival.

A+CHP: brentuximab vedotin, cyclophosphamide, doxorubicin, and prednisone.

CHOP: cyclophosphamide, doxorubicin, vincristine, and prednisone.

The response rates shown in the table are for only the ALCL patients.

Shading indicates studies done in pediatric while non-shaded are studies in adults.

In a phase II study, 102 patients with Hodgkin lymphoma who relapsed after autologous HSCT were treated with brentuximab vedotin at 1.8 mg/kg every 3 weeks (49). AEs were similar to the phase I study (peripheral sensory neuropathy, nausea, fatigue, neutropenia, and diarrhea). In a phase II study evaluating the safety and efficacy of brentuximab vedotin in patients with relapsed or refractory systemic ALCL, 58 patients were enrolled with a median age of 52 years (range 14 – 76 years) (43, 50). Sixteen were ALK^+^ (28%) while 42 were ALK^-^ (72%), and 29 (50%) had relapsed disease while the remaining 29 had refractory disease. Fifty of the 58 (86%) patients had an objective response with ALK^+^ and ALK^-^ disease having similar objective response rates (ALK^+^, 81%; ALK^-^, 88%). Thirty-eight (66%) of 58 patients achieved a CR, and these patients had a higher 5-year OS (79%) than those who did not (25%). Sixteen of the patients with a CR went on to either autologous (n = 8) or allogeneic (n =8) HSCT, with similar outcomes for each transplant type at five years. At the time of last follow-up, 16 of the 38 patients who achieved a CR remained in remission after receiving a median of 9 cycles of brentuximab vedotin. While 8 of these patients had received an HSCT (4 autologous, 4 allogeneic), 8 remained in remission without additional therapy. Though 33/58 (57%) of patients experienced peripheral neuropathy, 91% of these patients had resolution or improvement of their neuropathy at time of last assessment. This study demonstrated that brentuximab vedotin monotherapy is tolerable and effective in patients with relapsed/refractory ALCL and led to the FDA approval of brentuximab vedotin for adults with relapsed or refractory ALCL in 2011. Additional studies have demonstrated efficacy of brentuximab vedotin retreatment after initial brentuximab vedotin therapy (45). Eight patients with systemic ALCL had an objective response rate of 88% (63% CR) with toxicities similar to those described in other brentuximab monotherapy trials, though with higher rates of peripheral neuropathy given its cumulative toxic effect.

### Upfront therapy

The clinical success of brentuximab vedotin in patients with relapsed/refractory ALCL led to its use in newly diagnosed patients. A phase I study in patients with newly diagnosed CD30^+^ T-cell lymphoma evaluated brentuximab vedotin’s safety and activity when administered sequentially with CHOP or in combination with CHP (A+CHP) (46). Patients received either brentuximab vedotin monotherapy for two cycles followed by CHOP, or A+CHP for six cycles, with responders receiving an additional 8 – 10 cycles of brentuximab vedotin monotherapy. Thirty-two of the 39 patients studied had systemic ALCL with a median age of 57 years (range 21 – 82 years). For those who received sequential therapy (n=13), 11 (85%) had an objective response with 62% achieving a CR and a 1-year PFS of 77%. The sequential arm was terminated after observing progression of disease on CHOP after an initial response to brentuximab vedotin. Two of 13 (15%) patients experienced grade 3 – 4 peripheral sensory neuropathy. For those who received combination therapy (ALCL, n=19; non-ALCL, n=7), all 26 patients had an objective response with 88% achieving a CR and a 1-year PFS of 71%. After median 21.4 months, 11 (42%) had progressive disease or death. No patient went on to receive an HSCT. Toxicities while receiving combination therapy were manageable with grade 3 or higher AEs to include: febrile neutropenia (31%), neutropenia (23%), anemia (15%), pulmonary embolism (12%) and peripheral sensory neuropathy (8%). This study demonstrated that the combination therapy A+CHP is tolerable and effective in patients with ALCL.

The ECHELON-2 study formally tested the clinical efficacy and safety of A+CHP vs. CHOP. In this global, randomized phase III trial, previously untreated patients with CD30^+^ peripheral T cell lymphoma received either A+CHP or CHOP for 6 – 8 cycles (n=226 in each group) (47). The median age of participants was 58 years (range 18 – 85 years) and 80% had stage III – IV disease. Of the 452 patients, 316 (70%) had systemic ALCL, either ALK^+^ (n= 98) or ALK^-^ (n=218). Overall, both the OS and PFS were superior for the A+CHP arm (HR for OS=0.66, P=0.0244; HR for PFS=0.71, P=0.0110). The median PFS was 48.2 months (A+CHP) vs. 20.8 months (CHOP), with a 29% reduction in the risk of a PFS event. For those with systemic ALCL, there was a 41% reduction in the risk of a PFS event for those receiving A+CHP. This significant difference in outcomes persisted, with a 5-year PFS of 51.4% with A+CHP vs. 43.0% with CHOP (HR= 0.70; 95% CI: 0.53-0.91, P=0.0077) and a 5-year OS of 70.1% with A+CHP versus 61.0% with CHOP (HR=0.72; 95% CI: 0.53-0.99, P=0.0424) (51). This landmark trial demonstrated the superiority of A+CHP over CHOP and supported A+CHP as the new standard of care for adults with systemic ALCL.

## Clinical efficacy of brentuximab vedotin in children/adolescents

### Relapsed/refractory therapy

A multi-center phase I/II study evaluating brentuximab vedotin in pediatric patients with relapsed/refractory classical Hodgkin lymphoma or systemic ALCL enrolled 36 patients with a median age of 14 years (range 7 – 18 years) (48). Seventeen had ALCL, either ALK^+^ (n=12) or ALK^-^ (n=5), and six had already received a HSCT. This study determined the same recommended phase II dose as adults (1.8 mg/kg). Of the 17 patients with ALCL, 9 (53%) had a response and 7 (41%) patients achieved a CR at this dose. Patients with ALCL received a median of 9 cycles of therapy, and a total of 13 patients subsequently underwent an HSCT. The most common ≥ grade 3 AEs included neutropenia, increased γ-glutamyl transpeptidase, and pyrexia. Overall, 3/36 (8%) patients had a drug-related serious AE. While 12 of 36 (33%) patients experienced peripheral neuropathy, only one had severity ≥ grade 3 and 11 of the 12 cases improved or resolved. This trial was the first to demonstrate meaningful tumor response with a manageable safety profile in children/adolescents with CD30^+^ lymphoma receiving brentuximab vedotin monotherapy.

### Upfront therapy

The demonstrated safety and clinical efficacy of brentuximab vedotin provided compelling evidence to study it in newly diagnosed children with ALCL. The Children’s Oncology Group designed a randomized phase II clinical trial (NCT01979536) to determine the tolerability, EFS, and OS of adding either brentuximab vedotin or crizotinib to ALCL99 chemotherapy in children and adolescents with systemic ALK^+^/CD30^+^ ALCL (40, 52). Sixty-eight patients with a median age of 12 years (range 2-21 years) enrolled on the brentuximab vedotin arm of the study. Patients received brentuximab vedotin 1.8 mg/kg on day 1 of each 21-day cycle for a total of 6 cycles. All except three patients (94%) were able to receive the full dose of brentuximab vedotin in all 6 cycles. The 2-year EFS was 79.1% and the 2-year OS was 97.0% with no patient relapsing while receiving therapy. Like prior pediatric studies in ALCL, the major grade 3 – 4 toxicities included hematological events, mucositis, and febrile neutropenia. Importantly, there were no toxic deaths, no case of progressive multifocal leukoencephalopathy, and no case of grade 3 or 4 peripheral neuropathy. The addition of brentuximab vedotin prevented on-therapy relapses which have a poor prognosis. In addition, the EFS and OS compare favorably with all previous clinical trials making this regimen one of the standards of care for pediatric ALCL. For children who relapsed after receiving brentuximab vedotin as part of initial therapy, there are, unfortunately, no specific studies on the efficacy of rechallenging them with brentuximab vedotin.

### Brentuximab vedotin in cutaneous CD30^+^ disease

Specific subtypes of primary cutaneous T cell lymphomas (PCTL) are CD30^+^, including mycosis fungoides (MF), primary cutaneous ALCL (pcALCL), and lymphatoid papulosis (LyP). While these disorders can be more clinically benign and indolent than systemic CD30^+^ lymphomas, those that progress to a higher stage or systemic disease may have poor outcomes (53). Importantly, studies have demonstrated clinical efficacy of brentuximab vedotin in the treatment of CD30^+^ PTCL. A phase II study evaluating the safety and efficacy of brentuximab vedotin in treating 48 patients with CD30^+^ PCTL (28 MF, 2 pc-ALCL, 2 pc-ALCL plus LyP or MF, 9 LyP, and 7 LyP plus MF) at doses of 1.8 mg/kg every 21 days revealed an overall response rate of 73% (95% CI: 60 – 86%) and CR rate of 35% (95% CI: 22 – 49%) (53). In a phase III randomized, multi-center trial, patients with previously treated CD30^+^ mycosis fungoides (n=97) or pcALCL (n=31) were treated with either brentuximab vedotin (MF n=48; pcALCL n=16) or physician’s choice (MF n=49; pcALCL n=15) of either oral methotrexate or oral bexarotene. At median follow-up of 22.9 months, those with an objective global response lasting at least 4 months was 56.3% with brentuximab vedotin vs. 12.5% with physician’s choice (P<0.0001) (54). Response rates were higher for brentuximab vedotin over physician’s choice in MF (50% vs. 10.2%) and pcALCL (75% vs. 20%). These trials suggest clinical benefit in treating cutaneous CD30^+^ diseases with brentuximab vedotin.

## Discussion

The scientific advancements surrounding the understanding of ALCL biology and therapy have resulted in better outcomes for children/adolescents and adults with systemic ALCL. Brentuximab vedotin has demonstrated both safety and efficacy in the treatment of pediatric and adult systemic ALCL, and regimens with brentuximab vedotin are now the standard of care in newly diagnosed patients. Optimizing the use of brentuximab vedotin will continue to improve outcomes while hopefully reducing toxicity. Specifically, future studies should evaluate the role of brentuximab vedotin in combination with ALK inhibitors, which have also shown excellent activity in ALCL. Brentuximab vedotin in combination with immunotherapeutic strategies may also demonstrate therapeutic potential. For example, ALK^+^ ALCL tumors demonstrate strong PD-L1 expression, and the successful use of anti-PD1 monoclonal antibodies for ALCL patients has been reported in multiple cases (55–58). Brentuximab vedotin has already been successfully combined with checkpoint inhibitors in patients with Hodgkin Lymphoma and should be studied in ALCL as well. Finally, early studies of CD30-targeted chimeric antigen receptor (CAR) T cells in ALCL have demonstrated promising safety and activity in ALCL (59–61). These data suggest that there is great potential for combining brentuximab vedotin with other targeted therapies to treat ALCL and improve the survival and long-term quality of life of individuals affected by this rare disease.

## Author contributions

JA and EL: conceived of and designed the manuscript. JA: performed the literature search and drafted the manuscript. JA and EE: designed the tables/figures. JA, EE, EL: edited and revised the manuscript. All authors contributed to the article and approved the submitted version.
